# The Impact of Subclinical Hypothyroidism on Adverse Perinatal Outcomes and the Role of Thyroid Screening in Pregnancy

**DOI:** 10.3389/fendo.2019.00522

**Published:** 2019-08-06

**Authors:** Mei-Qin Wu, Jin Liu, Ya-Qian Wang, Ying Yang, Chong-Huai Yan, Jing Hua

**Affiliations:** ^1^MOE, Shanghai Key Laboratory of Children's Environmental Health, Xinhua Hospital Affiliated to Shanghai Jiao Tong University School of Medicine, Shanghai, China; ^2^Department of Nuclear Medicine, Shanghai Tenth People's Hospital, Tongji University, Shanghai, China; ^3^School of Public Health, Shanghai Jiao Tong University School of Medicine, Shanghai, China; ^4^The Women and Children's Health Care Department, Shanghai First Maternity and Infant Hospital, Tongji University School of Medicine, Shanghai, China

**Keywords:** subclinical hypothyroidism, pregnancy, hypertensive disorders of pregnancy, thyroid function, thyroid function screening

## Abstract

Subclinical hypothyroidism (SCH) is a mild form of hypothyroidism that is common among women of childbearing age. The impact of SCH on adverse perinatal outcomes is unclear and universal screening for thyroid function before or during pregnancy is also much debated. In the present retrospective cohort study on 7,587 women from Shanghai, we assessed whether SCH was associated with adverse perinatal outcomes. The relationship between the risks of adverse outcomes and the time of screening and LT4 treatment status for SCH were also evaluated. SCH was associated with hypertensive disorders of pregnancy (HDP) [odds ratio (OR): 4.04; 95% confidence interval (CI): 1.85–8.84; *P* = 0.000]. After classification into four different groups based on the time of screening for thyroid function, the increased likelihood of HDP persisted in those diagnosed with SCH in the first and second trimesters (OR: 9.69; 95% CI: 1.73–54.48; *P* = 0.01 and OR: 3.66; 95% CI: 1.07–12.57, *P* = 0.03, respectively). The diagnosis of SCH in the preconception period and the third trimester was not significantly associated with HDP and other adverse perinatal outcomes. Five out of 120 (5/120) treated women (4.17%) vs. 4/45 untreated women (8.89%) developed HDP, 4/5 were treated after conception. The results indicate that during pregnancy, SCH conferred an increased risk of HDP, particularly in women diagnosed with the disorder in the first and second trimesters.

## Introduction

Despite the well-known deleterious effects of overt thyroid dysfunction in women of reproductive age, the impact of subclinical hypothyroidism (SCH), a mild form of hypothyroidism defined as elevated thyroid stimulating hormone (TSH) greater than the upper limit of the reference range with normal free thyroxine (FT4) levels, on perinatal outcomes remains unclear (1, 2). On a worldwide scale, numerous studies with variable methodological quality have found inconsistent associations between SCH in pregnancy and adverse obstetrical outcomes including miscarriage, fetal death, preterm delivery, gestational diabetes, hypertensive disorders of pregnancy (HDP), placental abruption, low birth weight, 5 min Apgar scores <7 and lower IQ in childhood (3–6). The diagnostic criteria for SCH in pregnancy have changed over the years and vary between countries. These inconsistencies may be attributed to the differences in the definition of SCH (different TSH cut-offs), timing of TSH evaluation, and bias during enrolment of subjects and selection of end-point events (5, 7). Therefore, more studies including large samples and diverse populations are required to further evaluate the impact of SCH on prenatal outcomes.

To date, universal screening for thyroid dysfunction, both before and during pregnancy, is debated. The updated American Thyroid Association (ATA) guidelines published in 2017 concluded that there was insufficient evidence to make recommendations on the universal screening of thyroid dysfunction in the preconception phase or during early pregnancy (8). This may partly be due to the uncertainty regarding the impact of thyroid hormone replacement on maternal and neonatal outcomes. Two well-designed randomized controlled trials reported that maternal treatment for SCH did not result in improved cognitive function in their children (9, 10). However, since fetal thyroid hormones originate almost exclusively from the maternal system before 12–14 weeks of gestation, these results may have been influenced by delays in antenatal screening and initiation of treatment for hypothyroidism (in the second trimester) (11). Few studies have considered the impact of the timing of screening for SCH on perinatal outcomes. However, this is crucial for deciding the need and timing of routine screening of thyroid function in women who are trying to conceive.

In the present retrospective cohort study, we included 7,587 pregnant women to determine whether SCH is related to adverse perinatal outcomes. We also investigated the relationship between adverse perinatal outcomes and the timing of the first thyroid function test.

## Materials and Methods

### Study Population

**Figure 1:**
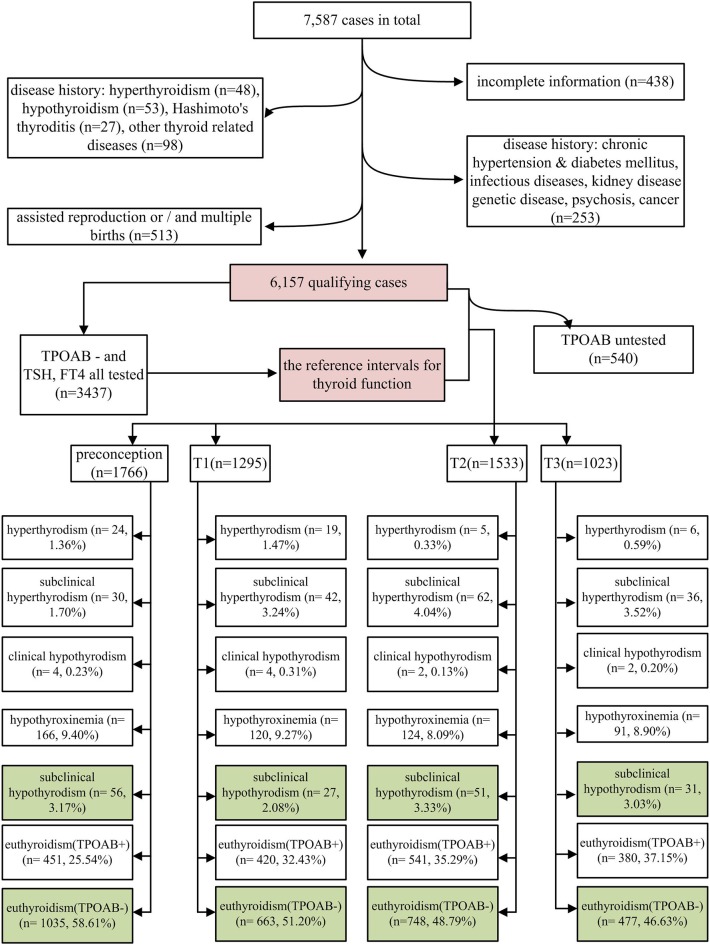
Flow diagram of study population determination process.

The study subjects were selected from a retrospective cohort study, which was conducted at the Shanghai First Maternity and Infant Hospital. The subjects were women who delivered at this hospital between January 1, 2015 and December 31, 2015. Each woman underwent thyroid function tests at least once during the 2 years prior to childbirth. A total of 7,587 women were identified as potential participants. A total of 438, 48, 53, 27, and 98 women with an incomplete history, hyperthyroidism, hypothyroidism, Hashimoto thyroiditis, and other thyroid diseases, respectively, were excluded in addition to 253 cases with a history of disease with possible impact on perinatal outcomes (including 10, 6, 3, 5, 12, 10, 201, and 6 cases with immune disorders, nephropathy, diabetes, hypertension, hereditary disease, cancer, infectious diseases, and other mental illness, respectively). Among the remaining 6,670 cases, 348, 290, and 125 cases of *in-vitro* fertilization (IVF), multiple births, and both IVF and multiple births, were excluded. Finally, 6,157 cases were eligible for analysis (Figure 1).

### Group Classification

According to the earliest time of thyroid function testing, the subjects were categorized into 4 different groups, namely preconception (within 2 years before pregnancy), first trimester (<13 weeks), second trimester ≥13 and <28 weeks), and third trimester (≥28 weeks).

### Trimester-Specific Reference Intervals of Thyroid Function

The present study established trimester-specific reference intervals of thyroid function according to the recommendations of the National Academy of Clinical Biochemistry (12) and the American Thyroid Association guidelines (8). The reference intervals of trimester-specific thyroid function are detailed in Tables S1, S2.

### Evaluation of Maternal Thyroid Function and LT4 Treatment Status

Maternal thyroid function was defined as any of the following: (1) overt hyperthyroidism: serum TSH <0.1 mIU/L and FT_4_ above the trimester-specific reference interval (97.5th percentile), excluding gestational hyperthyroidism; (2) subclinical hyperthyroidism: serum TSH concentration below the statistically defined lower limit of the trimester-specific reference range with serum FT_4_ concentrations within trimester-specific reference ranges; (3) overt hypothyroidism: TSH concentration above the trimester-specific reference interval (97.5th percentile), with a decreased FT_4_ (<2.5th percentile of the trimester-specific reference interval) or TSH concentration above 10.0 mIU/L irrespective of the level of FT_4_; (4) hypothyroxinemia: normal TSH concentration (between the 2.5th and 97.5th percentiles) in conjunction with FT_4_ concentrations in the lower 10th percentile of the reference range; (5) SCH: TSH higher than the 97.5th percentile and FT_4_ between the 2.5th and 97.5th percentiles; (6) euthyroidism with thyroid peroxidase antibody positivity (TPOAb+): both serum TSH and FT_4_ within the reference range (2.5th−97.5th) and anti-TPOAb levels higher than the upper limit of the reference value provided by the test kit (60 IU/mL in this study); and (7) euthyroidism with thyroid peroxidase antibody negativity (TPOAb–): both serum TSH and FT_4_ within the reference range (2.5th−97.5th) and anti-TPOAb levels lower than the upper limit of the reference value provided by the test kit. In current study, we focused on SCH; euthyroid and TPOAb– women were considered as the reference group (CON: labeled green in Figure 1). LT4 treatment status was identified from the medical history of the 165 SCH women.

### Evaluation of Adverse Perinatal Outcomes

The adverse perinatal outcomes were based on the diagnoses at discharge as per the medical records. We acquired data with a focus on the following adverse maternal and neonatal outcomes: gestational diabetes, hypertensive disorders of pregnancy (HDP), intrahepatic cholestasis of pregnancy (ICP), preterm labor, placenta previa, preterm premature rupture of membranes (PROM), fetal growth restriction (FGR), fetal distress, fetal death, low Apgar score (<7) at 5 min, low birth weight (birth weight <2,500 g), and macrosomia (birth weight >4,000 g). Factors that potentially impact the relationship between SCH and perinatal outcomes were chosen as potential confounders. We defined maternal age, gestational age, race, gravidity, parity, and delivery modality as possible confounding factors based on data from both, previous studies and our cohort.

### Statistical Analysis

All data were expressed as means ± standard deviations or numbers and percentages. The Student's *t*- and chi-squared tests were used to compare continuous and categorical variables, respectively. The risks of adverse outcomes in patients with SCH were determined by the chi-squared test and were presented as odds ratios (ORs) with 95% confidence intervals (CIs). After adjusting for confounders, multivariable logistic regression analysis was used to assess the associations between maternal SCH and obstetric outcomes. Statistical analysis was performed using the SPSS 19.0 software (SPSS Inc., Chicago, IL, USA) package. *P* < 0.05 was considered to be statistically significant.

## Results

### Characteristics of Participants

Among 6,157 eligible cases, the overall incidence of SCH was 2.68%; the highest and lowest incidences of SCH were observed in the second (3.33%; 51 of 1,533 cases) and first (2.08%; 27 of 1,295 cases) trimesters, respectively (Figure 1). Notably, abnormal thyroid function was detected in about 50% of the cases (Figure 1). In all stages of the perinatal period, euthyroidism with TPOAb positivity was found to be the most common type of thyroid dysfunction (25.54–37.15%), followed by hypothyroxinemia (8.09–9.40%) (Figure 1). Out of the 165 women with SCH, 120 received LT4 treatment and 45 did not. Therefore, 5/120 treated women (4.17%) vs. 4/45 untreated women (8.89%) developed HDP, and even from the treated women, 4/5 were actually started on treatment late in pregnancy, while only 1/5 was started before pregnancy.

The general demographic characteristics of the participants are shown in Table 1, categorized as the SCH (*n* = 165) and CON (*n* = 2,923) groups. Prior to grouping by different prenatal stages, race (*P* = 0.03) was found to be statistically different between the two groups (Table 1). After grouping according to the phases of the perinatal period, there were statistically significant differences between the groups with regard to race (*P* = 0.04) and parity (*P* = 0.03) in the preconception period. In the first trimester, gravidity (*P* = 0.001) was significantly different between the two groups. No differences were found in the general characteristics between the SCH and CON groups in both, the preconception and third trimester periods.

**Table 1:** Comparison of demographic Indexes between SCH and unexposed controls in different groups.

**Characteristics^**#**^**	**Total**			**Preconception**			**T1**			**T2**			**T3**		
	**SCH (*n* = 165)**	**CON (*n* = 2,923)**	***P***	**SCH (*n* = 56)**	**CON (*n* = 1,035)**	***P***	**SCH (*n* = 27)**	**CON (*n* = 663)**	***P***	**SCH (*n* = 51)**	**CON (*n* = 748)**	***P***	**SCH (*n* = 31)**	**CON (*n* = 477)**	***P***
Age(y), mean (SD)	30.26 (3.56)	30.36 (3.82)	0.74	30.52 (3.89)	30.23 (3.89)	0.59	29.22 (3.31)	30.18 (3.89)	0.20	30.22 (3.30)	30.60 (3.78)	0.48	30.77 (3.56)	30.50 (3.64)	0.68
Gestational age (d), mean (SD)	274.78 (9.75)	275.09 (9.66)	0.68	273.68 (9.84)	274.67 (9.90)	0.46	275.52 (9.06)	274.19 (10.61)	0.52	274.33 (11.04)	275.84 (8.82)	0.25	276.84 (7.78)	276.08 (8.83)	0.64
Race															
Han	159	2,880	0.03	52	1,014	0.04	27	654	–	50	741	0.48	30	471	0.36
Other	6	43		4	21		0	9		1	7		1	6	
Gravidity															
First	92	1,556	0.13	38	576	0.14	12	354	0.001	26	396	0.93	16	230	0.29
Second	36	831		13	281		4	209		14	207		5	134	
Third/higher	37	536		5	178		11	100		11	145		10	113	
Parity															
First	127	2,302	0.64	48	823	0.03	19	528	–	36	590	–	24	361	–
Second	36	602		6	204		8	130		15	156		7	112	
Third/higher	2	19		2	8		0	5		0	2		0	4	
Delivery model^*^															
Natural	105	1,878	0.62	31	672	0.34	15	442	0.39	36	464	0.56	23	300	0.20
C-section	56	1,001		24	346		11	211		14	273		7	171	
Forceps	4	43		1	17		1	10		1	11		1	5	

* *One of the women who tested thyroid function in the third trimester of pregnancy used the method of buttock-assisted delivery. #T1, first trimester; T2, second trimester; T3, third trimester*.

### Adverse Perinatal Outcomes in Women With SCH

The adverse perinatal outcomes in the SCH and CON groups are demonstrated in Table 2. No significant differences were found in the incidence of gestational diabetes, ICP, preterm labor, placenta previa, FGR, fetal distress, fetal death, Apgar score <7, birth weight <2,500 g, and birth weight more than 4,000 g. In contrast, HDP (5.45% vs. 1.47%; OR: 3.71; 95% CI: 1.84–7.48; *P* = 0.001) and PROM (22.42%% vs. 16.28%; OR: 1.38; 95% CI: 1.03–1.85; *P* = 0.04) were significantly higher in the SCH group. However, after controlling for confounding factors, statistical difference between the two groups was noted only for HDP (OR: 4.04; 95% CI: 1.85–8.84; *P* = 0.000).

**Table 2:** Adverse perinatal outcomes in women with SCH.

**Outcome**	**SCH (*n*, %)**	**CON (*n*, %)**	**Unadjusted**			Adjusted^*^		
			**OR**	**95% CI**	***P***	**OR**	**95% CI**	***P***
Gestational diabetes	25, 15.15%	342, 11.70%	1.30	0.89–1.88	0.18	1.05	0.0.66–1.69	0.84
HDP	9, 5.45%	43, 1.47%	3.71	1.84–7.48	0.001	4.04	1.85–8.84	0.000
ICP	2, 1.21%	21, 0.72%	1.69	0.40–7.13	0.47	1.72	0.40–7.42	0.47
Preterm labor	6, 3.64%	119, 4.07%	0.89	0.40–2.00	0.78	0.27	0.04–1.95	0.19
Placenta previa	9, 5.45%	104, 3.56%	1.53	0.79–2.97	0.21	1.53	0.76–3.09	0.23
PROM	37, 22.42%	476, 16.28%	1.38	1.03–1.85	0.04	1.41	0.96–2.05	0.08
FGR	0, 0.00%	12, 0.41%	–	–	–	–	–	–
Fetal distress	1, 0.61%	16, 0.55%	1.11	0.15–8.30	0.92	1.06	0.14–8.08	0.95
Fetal death	1, 0.61%	7, 0.24%	2.53	0.31–20.45	0.37	1.49	0.55–4.03	0.43
Apgar < 7	3, 1.82%	18, 0.62%	2.95	0.88–9.92	0.07	1.76	0.53–5.84	0.35
Birth weight < 2,500 g	2, 1.21%	80, 2.74%	0.44	0.11–1.79	0.24	0.43	0.11–1.77	0.24
Birth weight more than 4,000 g	11, 6.67%	156, 5.34%	1.25	0.69–2.26	0.46	1.28	0.68–2.41	0.44

* *Adjusted for race. HDP, hypertensive disorders of pregnancy; ICP, intrahepatic cholestasis of pregnancy; PROM, preterm premature rupture of membranes; FGR, fetal growth restriction*.

### Relationship Between Group-Specific SCH and Adverse Perinatal Outcomes

Based on the different stages of the perinatal period, SCH was not associated with an increased risk of pregnancy-related complications or adverse fetal growth outcomes in either the preconception or third trimester periods. However, in the first and second trimester, SCH was associated with HDP (OR: 9.69; 95% CI: 1.73–54.48; *P* = 0.01, and OR: 3.66; 95% CI: 1.07–12.57; *P* = 0.03, respectively) (Tables 3, 4). This indicates that pregnant women diagnosed with SCH in these stages were more likely to develop hypertension during the remainder of their pregnancy.

**Table 3:** Adverse perinatal outcomes in women diagnosed with SCH in the first trimester.

**Outcome**	**SCH (*n*, %)**	**CON (*n*, %)**	**Unadjusted**			Adjusted^*^		
			**OR**	**95% CI**	***P***	**OR**	**95% CI**	***P***
Gestational diabetes	4,14.81%	78,11.76%	1.26	0.50–3.19	0.55	1.23	0.41–3.67	0.71
HDP	2, 7.41%	5, 0.75%	9.82	2.00–48.36	0.001	9.69	1.73–54.48	0.01
ICP	0, 0.00%	5, 0.75%	–	–	–	–	–	–
Preterm labor	0, 0.00%	13, 1.96%	–	–	–	–	–	–
Placenta previa	3, 11.11%	25, 3.77%	2.95	0.95–9.16	0.09	3.36	0.94–12.06	0.06
PROM	5,18.52%	107,16.14%	1.15	0.51–2.58	0.79	1.29	0.47–3.50	0.62
FGR	0, 0.00%	4, 0.60%	–	–	–	–	–	–
Fetal distress	0, 0.00%	1, 0.15%	–	–	–	–	–	–
Perinatal mortality	0, 0.00%	1, 0.15%	–	–	–	–	–	–
Apgar < 7	0, 0.00%	4, 0.60%	–	–	–	–	–	–
Birth weight < 2,500 g	0, 0.00%	12, 1.81%	–	–	–	–	–	–
Birth weight more than 4,000 g	3, 11.11%	34, 5.13%	2.17	0.71-6.61	0.17	2.27	0.64-7.98	0.18

* *Adjusted for gravidity. HDP, hypertensive disorders of pregnancy; ICP, intrahepatic cholestasis of pregnancy; PROM, preterm premature rupture of membranes; FGR, fetal growth restriction*.

**Table 4:** Adverse perinatal outcomes in women diagnosed with SCH in the second trimester.

**Outcome**	**SCH (*n*, %)**	**CON (*n*, %)**	**Unadjusted**		
			**OR**	**95% CI**	***P***
Gestational diabetes	8, 15.69%	92, 12.30%	1.28	0.66–2.48	0.48
HDP	3, 5.88%	12, 1.61%	3.66	1.07–12.57	0.03
ICP	1, 1.96%	3, 0.40%	4.89	0.52–46.17	0.23
Preterm labor	4, 7.84%	47, 6.29%	1.25	0.47–3.33	0.68
Placenta previa	3, 5.88%	22, 2.95%	2.00	0.62–6.46	0.21
PROM	13, 25.49%	116, 15.51%	1.64	0.99–2.71	0.06
FGR	0, 0.00%	4, 0.54%	–	–	–
Fetal distress	0, 0.00%	4, 0.54%	–	–	–
Perinatal mortality	1, 1.96%	3, 0.40%	4.89	0.52–46.17	0.23
Apgar < 7	2, 3.92%	11, 1.47%	2.67	0.61–11.71	0.20
Birth weight < 2,500 g	1, 1.96%	27, 3.61%	0.54	0.08–3.92	1.00
Birth weight more than 4,000 g	4, 7.84%	42, 5.62%	1.40	0.52–3.74	0.53

*HDP, hypertensive disorders of pregnancy; ICP, intrahepatic cholestasis of pregnancy; PROM, preterm premature rupture of membranes; FGR, fetal growth restriction*.

## Discussion

### Main Findings

In the present study, the overall incidence of SCH was 2.68%. In total, 5/120 treated women (4.17%) vs. 4/45 untreated women (8.89%) developed HDP, and from the treated women 4/5 were actually started on treatment late in pregnancy. Except for race, the demographic indices were similar between the SCH and CON groups. SCH in pregnancy was associated with a significantly increased risk of HDP, particularly in those who were diagnosed with SCH in the first and second trimester. Conversely, in women who were tested for thyroid function before conception and the third trimester, a detrimental effect of SCH on the risk of maternal and neonatal outcomes was not observed.

### Data Interpretation and Comparisons to Findings in Previous Studies

The diagnostic criteria for SCH in pregnancy have changed over the years and vary between countries. Since the daily intakes of iodine, prevalence of thyroid autoimmunity, genetic backgrounds, and environmental factors vary between different populations, and the gestational age affects TSH levels, it is crucial to use a laboratory- or population-based trimester-specific TSH reference range for the diagnosis of SCH in pregnancy (8). Advances in the assessment of thyroid function have indicated that the interpretation of thyroid function tests depends on the stage of pregnancy (13). To facilitate precise evaluation of thyroid function during pregnancy, we used 4 different reference ranges for TSH and FT4 according to the 4 different perinatal stages. We also used population-derived 2.5th and 97.5th percentiles as reference intervals for the diagnosis of thyroid disorders, based on the overall analysis in this cohort. On the basis of these reference ranges, the incidence of SCH ranged from 2.08 to 3.33%. Studies using the same 97.5th percentile for the cut-off value for TSH as ours, have reported a similar prevalence of SCH (with an overall pooled-prevalence estimate of 3.47%) (1).

Reports suggest that SCH during pregnancy is associated with adverse perinatal outcomes, including miscarriage, preterm delivery, gestational diabetes, eclampsia, PROM, intrauterine growth restriction, and low birth weight (5, 14). Data from a few studies have also demonstrated that preconception SCH may be associated with a risk of infertility (15, 16). In the present study, we found a significant correlation between SCH and HDP; this finding concurs with that of many previous studies (11, 17, 18). However, published data have also suggested that SCH has no adverse impact on the outcomes of pregnancy (19, 20). The inconsistent results on the association between SCH and adverse pregnancy outcomes may be attributable to multiple factors, including the year the study was performed, the variable criteria used to identify the disease, and the gestational age at thyroid function screening.

HDP, including preeclampsia, gestational hypertension, chronic hypertension, and superimposed preeclampsia, has been shown to be a leading cause of maternal and perinatal morbidity and mortality in a multitude of large epidemiological studies worldwide (21–23). In our study, we observed that SCH was associated with HDP, particularly among women diagnosed with SCH in the first and second trimesters. However, the exact cause of HDP remains unknown. In addition to SCH, multiple factors including hormonal disorders, imbalances of angiogenic factors, and placental hypoxia also contribute to high blood pressure (24). In women with SCH, the mechanism for HDP may be based on decreased nitric oxide secretion and impairment of vasodilation in endothelial tissues (25). Hypercoagulability, increments in blood viscosity, and lipid abnormalities in patients with SCH potentially increase the risk for atherosclerosis; the increase of blood pressure in SCH may be related to these factors (26). However, the exact mechanism remains unclear.

In addition to the short-term impact on the mother and fetus, HDP also leads to an increase in the risk of other diseases later in life, including anxiety disorders in adolescent offspring and maternal cardiovascular diseases after pregnancy (27, 28). So early screening and treatment of SCH are both important. In our study, 5/9 women who developed HDP were intervened by LT4, but only one of them started before pregnancy. So it suggested that post-pregnancy treatment may reduce the chance for LT4 to have a beneficial effect. And the latest meta-analysis shows that LT4 replacement therapy can reduce blood pressure in SCH patients (29). But to date, the universal screening for thyroid function in childbearing women has been much debated in the scientific literature (6). Data on the role of thyroid screening on perinatal outcomes is limited, particularly for SCH. As the key parameter for SCH, the ATA guidelines of 2017 recommended screening for abnormal TSH concentrations in women planning assisted reproduction or known to have TPOAb positivity (8). Data on the role of thyroid screening in perinatal outcomes are limited, particularly with respect to SCH. Spencer et al. (30) suggested that compared with case finding or no screening, the universal screening of women before and during pregnancy was likely to be more effective in identifying women with thyroid dysfunction. In a large population-based cohort study in China, TSH elevation prior to conception was reportedly associated with an increased risk of adverse pregnancy outcomes, even in mild cases (7). Although the cases of SCH were not categorized in their study, the evidence suggests the need for perinatal screening of thyroid function; it also suggests that screening for TSH levels is particularly meaningful.

Compared to the adverse consequences in later life, screening for SCH in pregnancy is expected to be a cost-effective strategy. In the USA, analysis for cost-effectiveness has demonstrated that for every 100,000 pregnant women screened for SCH, $8,356,383 is saved, and 589.3 QALYs (marginal cost per quality-adjusted life year) are gained (31). Another study also reported that universal screening instead of high risk screening would result in an annual saving of €2,653,854 for the Spanish National Health System (32). The findings of this study suggest that if pregnancy is planned, universal screening should ideally be performed in the preconception period. However, in developing countries, the implementation of universal preconception thyroid screening may be hindered by economic constraints. The national preconception health examination project in China, which offers access to free preconception medical examinations, including thyroid hormone assays for rural couples planning a pregnancy, may be a good exemplary solution (33).

### Strengths and Limitations

The large sample size and the inclusion of women in the preconception period are some of the strengths of the present study. However, it also has some limitations. First, it was a retrospective study; therefore, the data analyzed were from women who had already delivered. Consequently, pregnancy loss caused by various factors (including SCH) before delivery was not considered during analysis. Second, the study focused on two time points, namely, that of the earliest thyroid function test and the time of delivery. Although we were able to identify the women who received LT4 treatment, detailed information (drug dosage and effect) on the specific interventions for SCH were not available, since at our center, most women with abnormal thyroid function are referred to endocrinology clinics for optimal treatment. Third, after grouping, the number of HDP cases in each SCH sub-group during pregnancy was very small; this may have reduced the power of the test. Further prospective trials with larger samples are needed to elucidate the relationship between SCH in the preconception period and perinatal outcomes in the Chinese population. The role of LT4 supplements also needs further evaluation.

## Conclusion

SCH during pregnancy, particularly when detected in the first and the second trimester, is associated with an increased risk of HDP, compared to euthyroid TPOAb- controls. Our findings suggest that routine thyroid function screening is necessary during pregnancy, particularly during the first and second trimesters.

## Data Availability

The datasets generated for this study are available on request to the corresponding author.

## Ethics Statement

This study complies with the guidelines of the Declaration of Helsinki. All data were processed anonymously.

## Author Contributions

M-QW, JL, Y-QW, YY, C-HY, and JH contributed to study design and acquisition of research data. M-QW and JL conducted the data analysis. M-QW drafted the manuscript. All authors contributed to improvements of the manuscript for important intellectual content and approved the final version for publication.

### Conflict of Interest Statement

The authors declare that the research was conducted in the absence of any commercial or financial relationships that could be construed as a potential conflict of interest.

## Abbreviations

CI: confidence interval
FGR: fetal growth restriction
FT_4_: free thyroxine
TPOAb+: thyroid peroxidase antibody positive
HDP: hypertensive disorders of pregnancy
ICP: intrahepatic cholestasis of pregnancy
IVF: *in-vitro* fertilization
LT_4_: levothyroxine
OR: odds ratio
PROM: preterm premature rupture of membranes
SCH: subclinical hypothyroidism
T1: first trimester
T2: second trimester
T3: third trimester
TPOAb–: thyroid peroxidase antibody negative
TSH: thyroid stimulating hormone.
