# Supplementary material for: The Impact of Subclinical Hypothyroidism on Adverse Perinatal Outcomes and the Role of Thyroid Screening in Pregnancy
Source: Front Endocrinol (Lausanne). 2019 Aug 6;10:522. doi: 10.3389/fendo.2019.00522 (PMC6691141; doi:10.3389/fendo.2019.00522)
Supplement: Supplementary file 1 [file Table_1.DOCX]

Table S1 Reference intervals (2.5^th^, 25^th^, median, 75^th^ and 97.5^th^ percentiles) for TSH (n =3437) in different time before delivery

| Time (group)^*^ | N | TSH（mIU/L） | | | | |
| --- | --- | --- | --- | --- | --- | --- |
|  |  | 2.5^th^ | 25^th^ | 50^th^ | 75^th^ | 97.5^th^ |
| Preconception | 1210 | 0.062 | 0.903 | 1.446 | 2.010 | 3.921 |
| T1 | 785 | 0.029 | 0.840 | 1.442 | 2.123 | 4.432 |
| T2 | 882 | 0.040 | 0.837 | 1.413 | 2.166 | 4.053 |
| T3 | 560 | 0.030 | 0.848 | 1.420 | 2.184 | 4.099 |

* T1: first trimester, T2: second trimester, T3: third trimester
