# Supplementary material for: The Impact of Subclinical Hypothyroidism on Adverse Perinatal Outcomes and the Role of Thyroid Screening in Pregnancy
Source: Front Endocrinol (Lausanne). 2019 Aug 6;10:522. doi: 10.3389/fendo.2019.00522 (PMC6691141; doi:10.3389/fendo.2019.00522)
Supplement: Supplementary file 2 [file Table_2.DOCX]

Table S2 Reference intervals (2.5^th^, 10^th^, 25^th^, median, 75^th^ and 97.5^th^ percentiles) for FT_4_ (n =3437) in different time before delivery

| Time (group)^*^ | N | FT4 (ng/dL) | | | | | |
| --- | --- | --- | --- | --- | --- | --- | --- |
|  |  | 2.5^th^ | 10^th^ | 25^th^ | 50^th^ | 75^th^ | 97.5^th^ |
| Preconception | 1210 | 0.930 | 1.010 | 1.080 | 1.170 | 1.250 | 1.507 |
| T1 | 785 | 0.917 | 0.996 | 1.070 | 1.160 | 1.255 | 1.590 |
| T2 | 882 | 0.920 | 1.010 | 1.090 | 1.180 | 1.320 | 1.571 |
| T3 | 560 | 0.930 | 1.020 | 1.090 | 1.200 | 1.360 | 1.651 |

* T1: first trimester, T2: second trimester, T3: third trimester
